# Inadequate pain management in prehospital emergency care: a retrospective study from Krapina-Zagorje County

**DOI:** 10.3325/cmj.2025.66.220

**Published:** 2025-06

**Authors:** Morena Milić, Ada Barić Grgurević, Josip Vrdoljak, Krunoslav Fučkar, Ana Brundula

**Affiliations:** 1 Department of Day Surgery, Dubrava University Hospital, Zagreb, Croatia; 2 University of Dubrovnik, Dubrovnik, Croatia; 3 Department of Anesthesia, Children's Hospital Srebrnjak, Zagreb, Croatia; 4 University of Split School of Medicine, Split, Croatia; 5 Department of Resuscitation and Intensive Care Medicine, Krapinske Toplice Special Hospital for Medical Rehabilitation, Zagreb, Croatia; 6 Polyclinic Amruševa, Zagreb, Croatia

## Abstract

**Aim:**

To assess the type of analgesics administered and their frequency of administration in pre-hospital pain management in Krapina-Zagorje County, Croatia.

**Methods:**

We retrospectively reviewed the data from 86 573 patients treated by the Emergency Department of Krapina-Zagorje County from October 1, 2017 to September 30, 2022. Data were obtained through the “e-Hitna” program. The inclusion criterion was a possible occurrence of pain based on the International Classification of Diseases diagnosis in patients who were treated by emergency team 1. We collected data on the type of analgesic and the frequency of administration according to age, the main diagnosis, emergency index, and the visual analog scale (VAS) score.

**Results:**

In 75% of the patients (64 938), analgesics were not administered. When analgesics were administered (21 635; 25%), physicians mostly opted for non-opioid analgesics (15 480, 71.6%). Opioid analgesia was usually the treatment of choice in palliative care. Tramadol was administered significantly more often than morphine or pethidine (*P* < 0.001, χ^2^ test).

**Conclusion:**

Despite wide accessibility of analgesics, pain is still not treated adequately in the Emergency Department of Krapina-Zagorje County.

The most recent definition of pain by the International Association for the Study of Pain is as follows: “An unpleasant sensory and emotional experience associated with, or resembling that associated with, actual or potential tissue damage.” (1). Pain is a common symptom in the pre-hospital emergency medicine setting. In a retrospective study from 2022, Ferri et al found pain prevalence in prehospital emergency medical services to range from 20% to 53% (2).

Moderate to severe pain is a frequent symptom in the prehospital setting, occurring in at least 28% of all acute patients transported by ambulance. The highest number of patients with moderate to severe pain suffer from injuries, non-specific diagnoses, and diseases of the circulatory system (3).

Although pain is common in patients managed by pre-hospital emergency teams, it is frequently not well treated. Jennings et al warn against oligoanalgesia, especially for pain of moderate and severe intensity (4). If untreated or improperly treated, pain may cause stress, endocrine and neurohumoral response, and a number of complications, negatively affecting organs and organ systems (5).

The Emergency Department of Krapina-Zagorje County, established in 2011, serves a population of 120 702 inhabitants and covers an area of 1229 km^2^ (6). The majority of inhabitants are rural residents. The Department consists of eight strategically distributed local offices. There are two emergency medical services teams: team 1, consisting of a physician, medical technician/nurse, and driver, and team 2, consisting of two medical technicians/nurses, who are trained in both medical skills and safe driving. Both teams are directed to the location by a dispatcher, who performs initial triage using bystander-obtained data. In Croatia, at the time of this research, the administration of drugs was allowed only to medical doctors, ie, only team 1 was allowed to administer drugs.

Data on quality indicators in health care are needed to understand how countries can improve their health service. Despite pain’s considerable effect on the quality of life, it has not been accepted as an indicator of the quality of health care. No previous studies in Croatia have comprehensively examined analgesic use across multiple International Classification of Diseases (ICD) groups in prehospital emergency care. Therefore, the aim of this study was to evaluate the types and frequency of analgesics used in prehospital pain management in Krapina-Zagorje County, Croatia.

## Patients and methods

We retrospectively reviewed the data on patients treated by the Emergency Department of Krapina-Zagorje County from October 1, 2017 to September 30, 2022. Medical documentation was obtained from the e-Hitna program (Rinels d.o.o., Rijeka, Croatia). The study was approved by the Ethics Committee of the Emergency Department of Krapina-Zagorje County.

The inclusion criterion was possible occurrence of pain based on the ICD diagnosis. The exclusion criterion was medical care provided by team 2, because at the time of the research in Croatia medical technicians/nurses were not allowed to administer drugs. After these criteria were applied, 86 573 patients remained in the sample. For these patients, we collected the data on the type of analgesic, the frequency of administration according to age, the main diagnosis, emergency index, and visual analog scale (VAS) score. Data on the VAS score, the ICD diagnosis, and the therapy applied are standard part of the Patient Medical Documentation Form (page A) for doctors in pre-hospital emergency teams in Croatia. The VAS score of 0 indicates the absence of pain, 1-3 indicates mild pain, 4-6 indicates moderate pain, and 7-10 indicates severe pain.

The call reception index for the medical reporting unit by the Croatian emergency service was modeled after the Norwegian emergency care index and distinguishes three levels of urgency: 1) the red priority (the designation A from the word “*akutt,*” meaning acute) – this category includes life-threatening conditions that require immediate medical intervention; 2) the yellow priority (the designation H from the word “*haster,*” meaning urgent, but not immediately life-threatening cases) – these situations require prompt medical attention and a doctor's examination, but they are not critical; 3) the green priority (designation V from the word “*vanlig,*” meaning regular) – this category includes conditions that do not require urgent medical care and can be managed with less immediate attention. The level of urgency is attributed to the patient's findings immediately after establishing telephone contact between the medical reporting unit and the patient.

### Statistical analysis

Data are presented as counts and percentages. For comparative analyses, the χ^2^ test of independence was employed to assess the association between categorical variables. The Fisher exact test was used for within-group comparisons of categorical variables when the sample sizes were small or when expected frequencies in any of the cells of a contingency table were below five. All statistical tests were two-tailed, and a *P* value of less than 0.05 was considered statistically significant. The Bonferroni correction was tailored to the number of comparisons in each analysis (eg, 3 for VAS categories, the number of ICD diagnosis groups, etc) to properly control for multiple testing across all tables. Statistical analyses were performed with Python (version 3.8), the scipy.stats module for statistical tests, and the matplotlib and seaborn libraries for data visualization.

## RESULTS

The total number of assessed patients was 105 944. Of these, 86 573 patients treated by team 1 were included in the final data set (46 753 or 54% were male). In 75% of the included patients (64,938), analgesics were not administered. When analgesics were administered (21,635, 25%), physicians mostly opted for non-opioid analgesics (15,480, 71.6%). Among patients who were not administered analgesics, 43.4% were not assessed with the VAS (28,210), 40% did not report pain (25,974), and 16.6% were not asked about pain (10,754).

Most patients were diagnosed with diseases of the circulatory system (I00-99 ICD), followed by those who suffered injuries and poisoning (S00-T98), had neoplasms (C00-D48), and received palliative care (Z51.5). The ICD groups significantly differed in terms of analgesics administration (*P* < 0.001, χ^2^ test; Table 1). The *post-hoc* in-between group comparison also showed a significant difference for each pair (*P* < 0.001, Fisher exact test). All patients in the musculoskeletal system and connective tissue group (M00-M99), 60.32% of those diagnosed with neoplasms (C00-D48) or receiving palliative care (Z51.5), and only 18.74% of patients in the injuries and poisoning group (S00-T98) received analgesics.

**Table 1:** Distribution of analgesics administration per the International Classification of Diseases (ICD) group

	Total	Patients in whom analgesics were not administered		Patients in whom analgesics were administered		P (Χ^2^ test)
ICD group		number	%	number	%	
Neoplasms (C00-D48) and palliative care (Z51.5)	14 978	5943	39.66	9035	60.34	
Diseases of the circulatory system (I00-I99)	33 937	31 060	91.52	2877	8.48	
Diseases of the digestive system (K00-K99)	4415	3754	85.04	661	14.96	
Diseases of the musculoskeletal system and connective tissue (M00-M99)	1982	0	0.00	1982	100.00	
Diseases of the genitourinary system (N00-N99)	1770	885	50.00	885	50.00	
Symptoms, signs and abnormal clinical and laboratory findings (R00-R99)	11081	8337	75.25	2744	24.75	
Injury, poisoning (S00-T98)	18 410	14 959	81.26	3451	18.74	
Total	86 573	64 938	74.98	21 635	25.02	<0.001

The ICD groups significantly differed in terms of opioid administration (Χ^2^ test, *P* < 0.001; Table 2). The *post-hoc* in-between group comparison also showed a significant difference for each pair (*P* < 0.001, Fisher exact test). Opioids were most commonly used in patients with neoplasms (C00-D48) and those who received palliative care (Z51.5), accounting for 54.92% of cases, followed by those with injuries and poisoning (S00-T98) (33.35%). In terms of specific opioid drugs, tramadol was administered significantly more often than morphine or pethidine (*P* < 0.001, Χ^2^ test)

**Table 2:** Distribution of opioid analgesics per the International Classification of Diseases (ICD) group

	Total	Patients taking non-opioid analgesics		Patients taking opioid analgesics		P (Χ^2^ test)
**ICD group**		**number**	**%**	**number**	**%**	
Neoplasms (C00-D48) and palliative care (Z51.5)	9035	4073	45.08	4962	54.92	
Diseases of the circulatory system (I00-I99)	2877	2856	99.27	21	0.73	
Diseases of the digestive system (K00-K99)	661	657	99.39	4	0.61	
Diseases of the musculoskeletal system and connective tissue (M00-M99)	1982	1979	99.85	3	0.15	
Diseases of the genitourinary system (N00-N99)	885	884	99.89	1	0.11	
Symptoms, signs, and abnormal clinical and laboratory findings (R00-R99)	2744	2730	99.49	14	0.51	
Injury, poisoning (S00-T98)	3451	2301	66.65	1150	33.35	
Total	21 635	15 480	71.56	6155	28.44	<0.001

**Figure 1:**
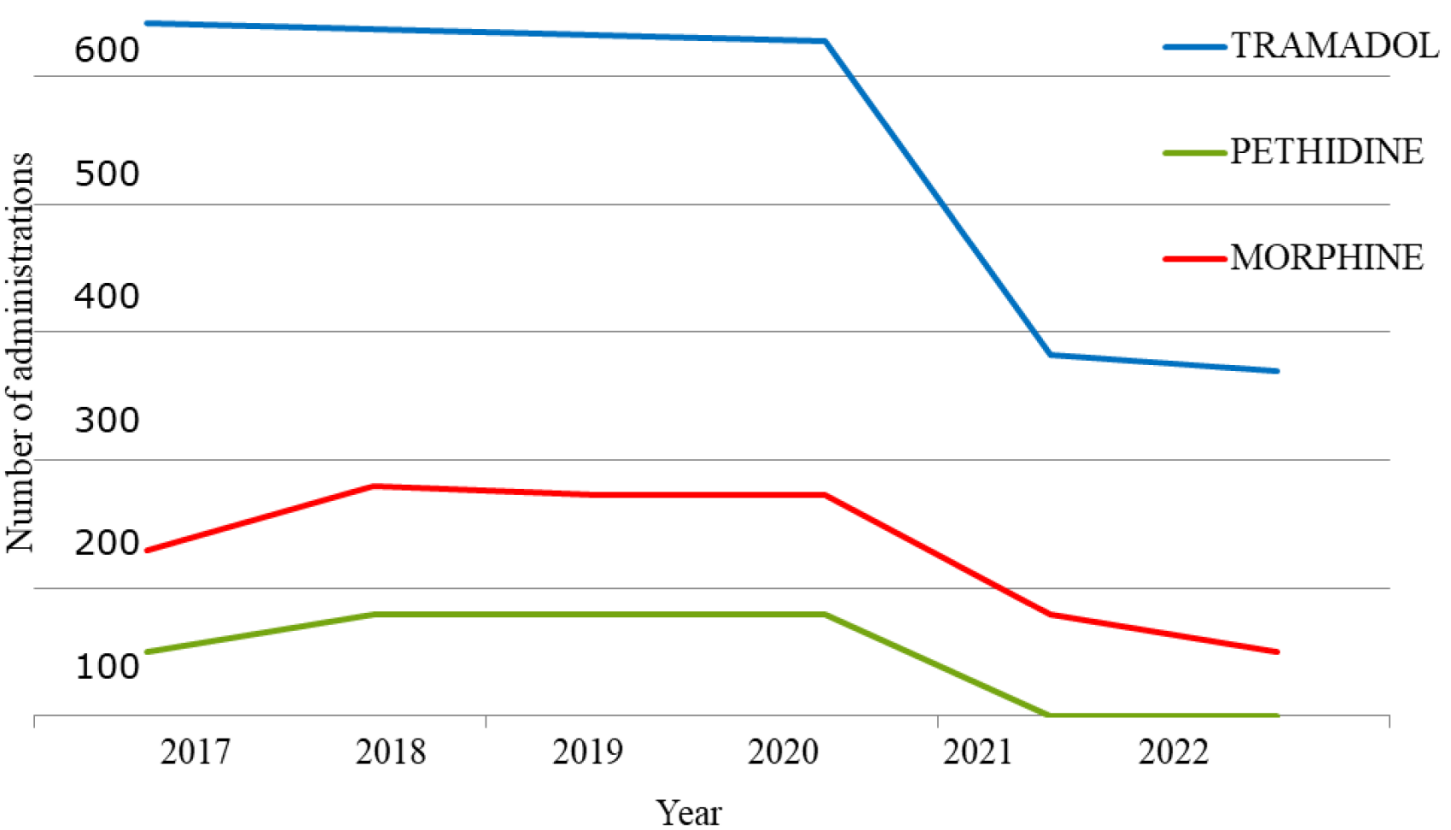
Opioid pain management in Krapina-Zagorje County between 2017 and 2022.

Only 0.008% of the children in our study received pain treatment, predominantly with non-steroidal analgesics. Opioids were administered in only one case – a child with burn injuries. In the entire sample, the use of analgesics clearly declined during the COVID pandemic (Figure 1).

In patients reporting mild to moderate pain (VAS scores 1-3 and 4-7), non-opioid analgesics were administered significantly more often than opioids (*P* < 0.0001, Χ^2^ test). In the VAS 8-10 category, opioid analgesics were administered more often (*P* < 0.001). Among them, tramadol was again the most common choice (Table 3). Furthermore, opioids were administered significantly more often in the >65 age group than in 20-65, 7-19, and 0-6 age groups (*P* < 0.001, Χ^2^ test and Fisher exact test) (Table 4). They were also administered significantly more frequently in patients from the “V” emergency index category than in patients from the “H” and “A” categories (*P* < 0.001, Χ^2^ test and Fisher exact test; Table 5).

**Table 3:** Distribution of analgesics per visual analog scale (VAS) score

	Total	Non-opioid analgesics		Opioid analgesics (total)		Tramadol		Morphine		Pethidine	
VAS score		n	%	n	%	n	%	n	%	n	%
1-3	2812	2618	93.10	194	6.90	194	6.90	0	0.00	0	0.00
4-7	10 169	9515	93.57	654	6.43	636	6.25	16	0.16	2	0.02
8-10	8654	3347	38.67	5307	61.33	4011	46.35	970	11.21	326	3.77
**Total**	**21 635**	**15 480**	**71.56**	**6155**	**28.44**	**4841**	**22.38**	**986**	**4.56**	**328**	**1.52**

**Table 4:** Pain management according to the age group

	No pain management		Analgesics		Non-opioid analgesics		Opioid analgesics	
Age group (years)	n	%	n	%	n	%	n	%
0-6	5925	99.19	49	0.81	48	98.0	1	2.0
7-19	8993	92.15	703	7.85	698	99.3	5	0.7
20-65	17 919	25.12	4503	74.88	2972	66.0	1531	34.0
>65	32 101	50.99	16 380	49.01	11 762	71.8	4618	28.2
Total	64 938	75.06	21 635	24.94	15 480	71.56	6155	28.44

**Table 5:** Opioid analgesics used according to the emergency index level

	Total	Tramadol		Morphine		Pethidine	
Emergency index (urgency level)*		n	%	n	%	n	%
V	4983	3692	74.09	964	19.35	327	6.56
H	407	405	99.51	2	0.49	0	0.00
A	765	744	97.25	20	2.61	1	0.13
Total	6155	4841	78.65	986	16.02	328	5.33

*A – life-threatening emergency requiring immediate intervention (highest priority); H –urgent condition requiring prompt care but not immediately life-threatening; V – non-urgent cases where delayed care is acceptable.

## DISCUSSION

Adequate pain management should be an integral part of patient care. Unfortunately, in our study, pain was not routinely treated and was often underestimated – analgesia was used only in a quarter of our patients. Pain is an individually perceived feeling and, in addition to the sensory component, it is affected by an emotional and cognitive component (7). Moreover, even 16.6% of our respondents were not asked about pain. Although simple visual analog and numerical scales are available for pain assessment, in our study, they were insufficiently used.

Mašala et al (8) studied the incidence and treatment of pain associated with malignant diseases in emergency medicine in Zagreb. They also showed that one-dimensional scales were not used in daily clinical practice to assess the intensity of pain as a basis for the prescription of analgesic therapy (8).

According to our results, pain was most frequently treated by non-opioid drugs (71.6%). Pain management with non-opioid analgesics should not be the first choice as the majority of patients included in the study belonged to the ICD categories where moderate (VAS 4-7) to severe pain (VAS 8-10) is expected: ischemia, trauma, and malignant diseases.

When analyzing the type of analgesic administered, opioids were most often used in patients with neoplasms (C00-D48) and those receiving palliative care (Z51.5), followed by patients in the injuries and poisoning group (S00-T98). At the same time, they were used only in 0.007% of patients with diseases of the circulatory (cardiovascular) system (I00-99), which includes patients with suspected acute coronary syndrome and pulmonary edema.

Tramadol, a weak opioid, was the most commonly used opioid among our patients. Morphine and pethidine were used more frequently when pain was severe (VAS 8-10) than when it was mild or moderate (VAS 2-7). However, tramadol remained the most common drug of choice regardless of pain severity or cause. Mašala et al (8) also observed the predominance of non-opioid analgesics (58.72%), followed by tramadol (39.74%), and only sporadic use of strong opioids (1.55%) in patients with malignant pain managed by pre-hospital emergency medicine services in Zagreb.

Kosiński et al (9) determined the frequency of pain in patients with different medical conditions treated by pre-hospital emergency medicine. They observed insufficient pain treatment, especially when it came to severe pain, as evidenced by the sporadic use of opioids. Of all the pain-reducing drugs, the most commonly used was ketoprofen (9).

In patients with malignant diseases in whom opioid therapy was indicated, severe pain was often not recognized and therefore not adequately treated. According to Bossi et al, despite existing guidelines, many patients did not receive adequate breakthrough pain treatment, which adversely affected their quality of life (10).

Injuries are the leading cause of hospitalization and death in children in Croatia (11). Our findings are worrying because they indicate a striking absence of knowledge of pain treatment in children. Researchers have been warning about poor pre-hospital analgesia for children. For example, Izsak et al observed insufficient pre-hospital pain relief in 83% of pediatric patients (12).

In our research, the largest number of patients who received opioids belonged to the emergency index “V” category, ie, the lowest level of urgency. This is understandable given that precisely patients with neoplasms and those receiving palliative care, who are classified in the category “V,” were among the more frequent callers of the pre-hospital emergency medicine services.

The COVID-19 pandemic significantly changed the functioning of the health care system in Croatia. The pandemic imposed additional demands on a system that was already understaffed. For this reason, the employees became anxious and afraid of infection and transmission of the coronavirus to the family members (13). The decrease in analgesic use during the pandemic observed in this study could be linked to the heightened stress and operational challenges faced by health care professionals.

Effective treatment of acute pain consists of ethical and humane procedures, which, according to our research, are not sufficiently integrated into the daily work of pre-hospital emergency services. A possible reason for this could be fear of using opioids or just ignorance. Fear of using opioids may be explained by the problems associated with opioid abuse, as exemplified by the opioid crisis in the US (14). However, a 2021 study on the use of opioid analgesics in 25 European countries showed that Europe was not facing an opioid crisis, and the use of opioids was lower in the countries of Eastern Europe than in Western and Northern Europe (15). The number of opioid-related deaths in Croatia (2015 to 2017) and Germany (2017 to 2019) was stable (15). This finding indicates that discussions on the potential harms of opioids should not prevent their prescription for cancer pain and palliative care. Unlike some Eastern European countries (15), opioid analgesics are widely available in Croatia, which removes any obstacles for adequate pain management.

The current study suffers from several limitations. A considerable proportion of patients (43.4%) did not undergo pain validation using the VAS, which may have affected the accuracy of pain assessment and subsequent decisions regarding analgesic administration. Additionally, information on analgesic allergies was unavailable through the automated search of the e-Hitna database, preventing us from including these data in the analysis. Selection bias is evident, as 75% of patients did not receive analgesics, and many had no documented pain validation. As a result, the findings primarily reflect the cases when analgesics were administered, potentially skewing the analysis. Also a limitation of this study is its single-center design, as data were collected only from the Emergency Department of Krapina-Zagorje County. Therefore, caution should be exercised when extrapolating these results to the entire Croatian health care system or to other countries. Differences in regional health care resources and patient populations may affect the generalizability of the findings. Further multicenter studies across diverse settings are needed to validate and expand these results.

In conclusion, our results indicate that despite the high prevalence of pain in pre-hospital treatment and the wide accessibility of analgesics, pain is still not treated adequately in Zagorje-Krapina County. Improved education, defining, and implementing pain management protocols and individual knowledge self-assessment are necessary for pain to be recognized, measured, and treated successfully.
